# Analysis of association between low birth weight and socioeconomic deprivation level in Japan: an ecological study using nationwide municipal data

**DOI:** 10.1186/s40748-022-00143-z

**Published:** 2022-10-06

**Authors:** Tasuku Okui, Naoki Nakashima

**Affiliations:** Medical Information Center, Kyushu University Hospital, Fukuoka city, Japan

**Keywords:** Japan, Low birth weight, Municipalities, Socioeconomic status, Vital statistics

## Abstract

**Background:**

Several international studies have indicated an association between socioeconomic deprivation levels and adverse birth outcomes. In contrast, those investigating an association between socioeconomic status and low birth weight using nationwide data are limited in Japan. In this study, we investigated an association between municipal socioeconomic deprivation level and low birth weight by an ecological study.

**Methods:**

Nationwide municipal-specific Vital Statistics data from 2013 to 2017 were used. We calculated the low birth weight rate and standardized incidence ratio (SIR) for low birth weight for each municipality and plotted them on a Japanese map. Furthermore, the correlation coefficient between them and the deprivation level were calculated. In addition, a spatial regression model including other municipal characteristics was used to investigate an association between low birth weight and the deprivation level.

**Results:**

Municipalities with relatively high SIR for low birth weight were dispersed across all of Japan. The correlation coefficient between the socioeconomic deprivation level and low birth weight rate was 0.196 (*p*-value < 0.001) among municipalities, and that between the socioeconomic deprivation level and the SIR for low birth weight was 0.260 (*p*-value < 0.001). In addition, the spatial regression analysis showed the deprivation level was significantly and positively associated with low birth weight.

**Conclusions:**

The socioeconomic deprivation level and low birth weight were positively associated, and a further study using individual data is warranted to verify reasons for the association.

## Background

Worldwide, the prevalence of low birth weight is estimated to have decreased from 2000 to 2015 [1]. In contrast, the prevalence of low birth weight had increased over the past decades in Japan [2], and has only begun to decrease in recent years [3]. Low birth weight in infants is a representative adverse birth outcome. Additionally, low birth weight is a major risk factor associated with infant mortality and neonatal mortality [4, 5], and is also associated with the incidence of diabetes or cardiovascular disease in adulthood [6]. Moreover, low birth weight has been shown to be related to height in adulthood [7]. Therefore, there is the need to further reduce its incidence.

Among the risk factors for low birth weight is socioeconomic status. Studies have reported that lower socioeconomic status is associated with a higher prevalence of low birth weight in infants [8, 9]. In contrast, studies investigating the socioeconomic status and the rate of low birth weight using nationwide data are relatively limited in Japan. Previously, an epidemiological study showed that high household income was positively associated with the risk of low birth weight [10], with the tendency to be lean among women with high socioeconomic status being pointed out as a possible reason for the association [10]. Another previous study using nationwide data showed that the risk of low birth weight was higher in mothers who were manual workers [11], but it was not certain whether the manual work itself had a negative effect on low birth weight or if other kinds of lower socioeconomic status, such as lower educational levels, were more responsible for the higher rate. It will be meaningful to investigate an association between low birth weight and socioeconomic status using additional socioeconomic indicators and to reveal whether this disparity also exists in Japan.

Area-level socioeconomic deprivation is an indicator which is used for investigating differences in health outcomes depending on areal-based socioeconomic status. There are many studies indicating an association between area-level socioeconomic deprivation and adverse outcomes in other countries [12–17], and regional disparities have also been revealed. However, there have been no studies investigating this association in Japan. In addition, there have been no studies investigating regional differences in rates of low birth weight using nationwide municipal-level data in Japan, while there have been some studies investigating differences in the rate among prefectures [18–20]. Therefore, municipalities with high low birth weight rate are unknown. By focusing on differences in the rate of low birth weight depending municipal socioeconomic deprivation level, an association might be able to be identified more precisely, and more effective preventive measures targeting a municipality can be implemented.

In this study, we investigated an association between municipal socioeconomic deprivation level and low birth weight rate using nationwide municipal-specific data.

## Methods

### Data

Low birth weight infants are defined as infants whose birth weight are less than 2,500 g. Aggregate data on numbers of low birth weight infants and births for each municipality during the period 2013–2017 were obtained from the Specified Report of Vital Statistics [21]. Additionally, the number of births by maternal age group for each municipality, and the number of low birth weight infants by maternal age group from 2013 to 2017 were obtained from the national Vital Statistics [22].

As an indicator of municipal socioeconomic deprivation level, an indicator derived from multiple socioeconomic characteristics of municipalities was used [23]. Principal component analysis was usually used for obtaining areal social deprivation levels [23–25], and the socioeconomic deprivation levels are derived by summarizing those socioeconomic characteristics using principal component analysis. The deprivation level derived in a previous study can be calculated with the following equation using standardized values for all the characteristics [23].

Socioeconomic deprivation level = 0.591 $\times$ proportion of divorced persons + 0.517 $\times$ proportion of fatherless households + 0.470 $\times$ proportion of unemployed persons + 0.201 $\times$ proportion of laborers + 0.110 $\times$ proportion of low educational level − 0.152 $\times$ proportion of households living in owner–occupied housing − 0.293 $\times$ taxable income per capita.

In addition, other municipal characteristics that are possibly associated with regional differences in low birth weight rate were used in the analysis. Specifically, population density, number of births per capita, proportion of women aged 15–49, number of physicians working in an obstetrics and gynecology department per capita, number of medical clinics per capita, and number of hospitals per capita. Physicians working in an “obstetrics and gynecology” department include those working in an obstetrics department. Data on municipal socioeconomic characteristics and other characteristics were obtained from Census data, the survey on taxation status of municipal taxes, the Survey of Medical Institutions, the Statistics of Physicians, Dentists and Pharmacists, the Municipalities Area Statistics of Japan, and the population, demographics, and household numbers based on the basic resident register published by the Ministry of Internal Affairs and Communications [26–29]. Regarding those municipal characteristics, all the data in 2015 were extracted, but data on number of physicians working in an obstetrics and gynecology department in 2014 and data on proportion of low educational level in 2010 were used because the survey for those characteristics was not conducted in 2015.

Moreover, map data of Japan were obtained from the digital national land information published by the Ministry of Land, Infrastructure, Transport, and Tourism, and were further processed by the authors [30].

### Statistical analysis

As part of the statistical analyses, the low birth weight rate was calculated for each municipality using data on the number of low birth weight infants and total births. There were regional differences in maternal age distribution among municipalities, and an analysis was conducted taking into account maternal age. First, the low birth weight rate for each maternal age group was calculated for all of Japan. For this analysis, publicly available aggregate data grouped by maternal ages (less than 15 years old, 15–19 years old, 20–24 years old, 25–29 years old, 30–34 years old, 35–39 years old, 40–44 years old, 45 years old or more) were used, after which we conducted an analysis based on those maternal age groups. Then, the number of births was multiplied with the low birth weight rate in Japan for each maternal age group and municipality, and the expected number of low birth weight infants for each maternal age group and municipality was calculated. By summing the expected number of low birth weight infants for all maternal age groups, the expected number of low birth weight infants was calculated for each municipality. Therefore, the expected number of low birth weight infants for each municipality $i$ (${\mathrm{The expected number}}_{i}$) was calculated based on the following equation:

$${\mathrm{The expected number}}_{i}={\sum }_{j=1}^{J}{\mathrm{low birth weight rate}}_{j}{\times \mathrm{Number of births}}_{ij}$$,

where $j$ indicates each maternal age group, $J$ is the total number of maternal age groups, ${\mathrm{low birth weight rate}}_{j}$ indicates the low birth weight rate for maternal group $j$ in Japan, and ${\mathrm{number of births}}_{ij}$ indicates the number of births for each maternal group $j$ in municipality $i$. With the actual and expected numbers of low birth weight infants, the standardized incidence ratio (SIR) for low birth weight was also calculated for each municipality using the empirical Bayes method, as conducted in previous studies [31, 32]. Specifically, the actual numbers of low birth weight infants were supposed to follow the Poisson distribution, whose mean was SIR × the expected numbers of low birth weight infants when calculating the SIR [33, 34]. SIR is almost the same as the ratio of the actual number of low birth weight infants to the expected number of low birth weight infants for each municipality.

Subsequently, an ecological study was conducted to investigate an association between low birth weight births and the socioeconomic deprivation level using municipal-level data. The low birth weight rate and the SIR were plotted onto a Japanese map to show geographical differences in low birth weight rates across Japan. In addition, a scatterplot between the deprivation level and the SIR for low birth weight was shown, and the Pearson correlation coefficient between them was calculated. Moreover, the municipalities were classified into quintiles, and summary statistics of low birth weight rate and other municipal characteristics were calculated for each quintile.

Poisson regression model was applied using the number of low birth weight infants as the response variable and the socioeconomic deprivation level and other municipal characteristics as explanatory variables. The expected number of low birth weight infants was used as an offset term in the Poisson regression model in order to adjust for the difference in number of births and maternal ages among municipalities. However, spatial autocorrelation of the outcomes is usually observed. Therefore, Moran’s I statistics for the residuals of the Poisson regression analysis were calculated in order to measure for spatial autocorrelation. Bayesian spatial regression modeling was also used to investigate the association and to account for spatial autocorrelation [35]. In the Bayesian spatial regression model, the number of low birth weight infants was also supposed to follow a Poisson distribution. Relative risk, 95% confidence intervals, and *p* values were calculated for the Poisson regression model, and the posterior mean of relative risk for low birth weight and 95% credible intervals were calculated in the Bayesian spatial regression model. All the explanatory variables were scaled when applying a regression model. Because a spatial model was used, municipalities on islands which were not adjacent to another municipality were not used in the ecological analysis. Additionally, six municipalities whose populations in 2015 were extremely small because of evacuations were not used in the ecological study. All statistical analyses were conducted using R, ver.4.1.3 (https://www.R-project.org/).

An approval by institutional review board was not required because only data that are publicly available were used.

## Results

There were 4,962,247 births and 471,050 low birth infants in total in Japan from 2013 to 2017.

Table 1 shows the number of births and the low birth weight rate in Japan by maternal age group and year. Although the number of births was high in mothers 25–39 years, the low birth weight rate was particularly higher in mothers 45 years or more.

**Table 1:** Number of births and Low birth weight rate in all of Japan by maternal age group and year

	Maternal age groups						
Year	< 20 years	20–24 years	25–29 years	30–34 years	35–39 years	40–44 years	> 44 years
Number of births							
2013	12,964	91,251	282,778	365,381	229,728	46,544	1,116
2014	13,010	86,589	267,833	359,293	225,873	49,603	1,272
2015	11,930	84,465	262,259	364,868	228,282	52,554	1,308
2016	11,099	82,194	250,709	354,984	223,310	53,478	1,401
2017	9,900	79,272	240,949	345,414	216,947	52,101	1,512
Low birth weight rate^*^							
2013	102.6	90.0	88.3	92.5	105.4	125.9	214.2
2014	100.8	89.0	87.5	92.5	105.1	122.9	203.6
2015	99.9	89.8	86.7	91.3	103.7	123.0	176.6
2016	100.6	88.0	86.4	92.0	101.6	121.6	187.7
2017	106.5	89.9	88.0	90.8	101.6	121.5	167.3

^*^Number of low birth weight infants per 1,000 births

**Fig. 1:**
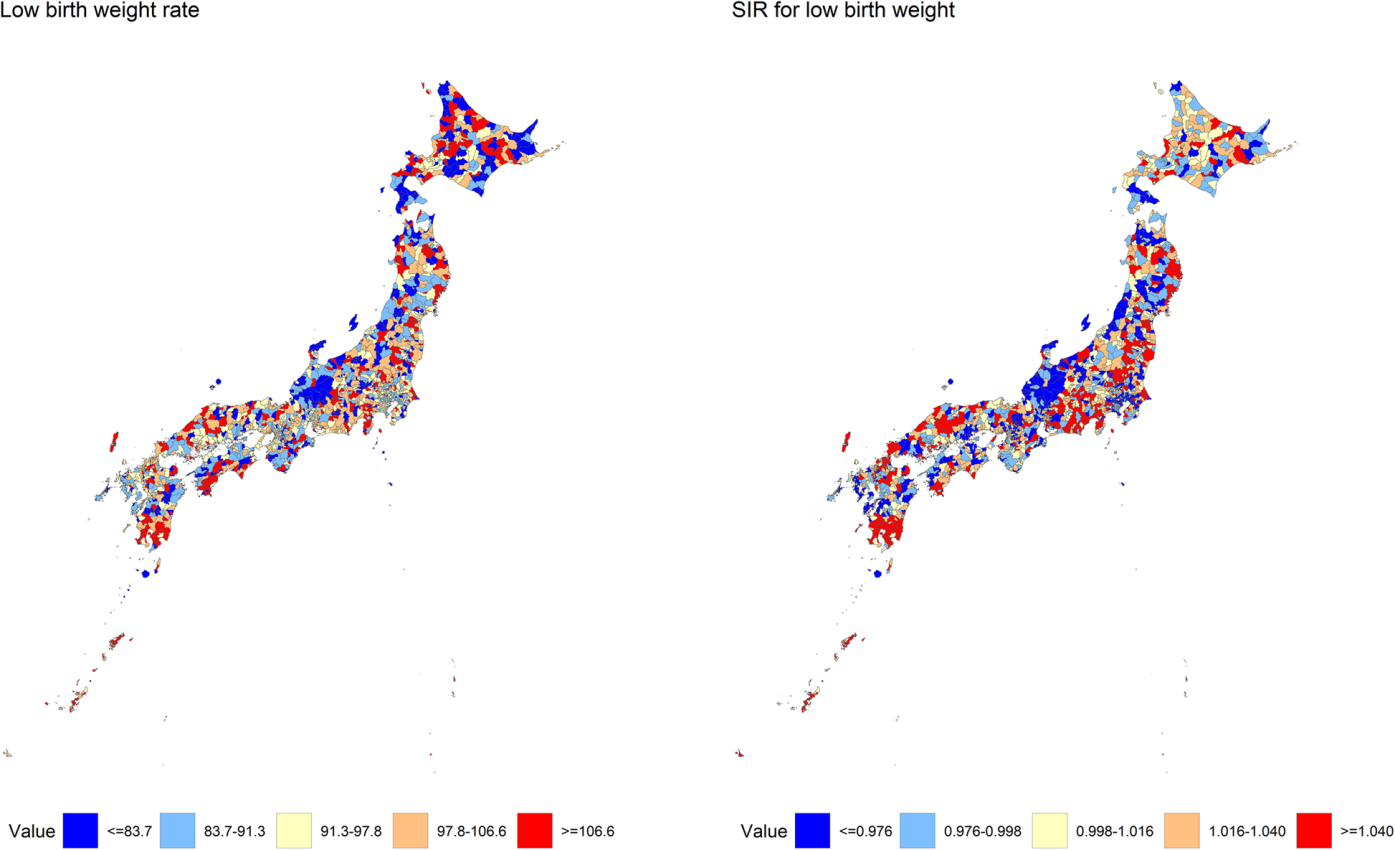
Geographic differences in low birth weight rate and SIR for low birth weight in Japan. SIR, standardized incidence ratio. Low birth weight rate indicates the number of low birth weight infants per 1,000 birth years

Figure 1 shows the geographic difference in low birth weight rate and SIR for low birth weight in Japan. Municipalities with relatively a high low birth weight rate or the SIR were dispersed across the country. High SIRs tended to be observed, particularly in Kagoshima and Okinawa Prefectures.

**Fig. 2:**
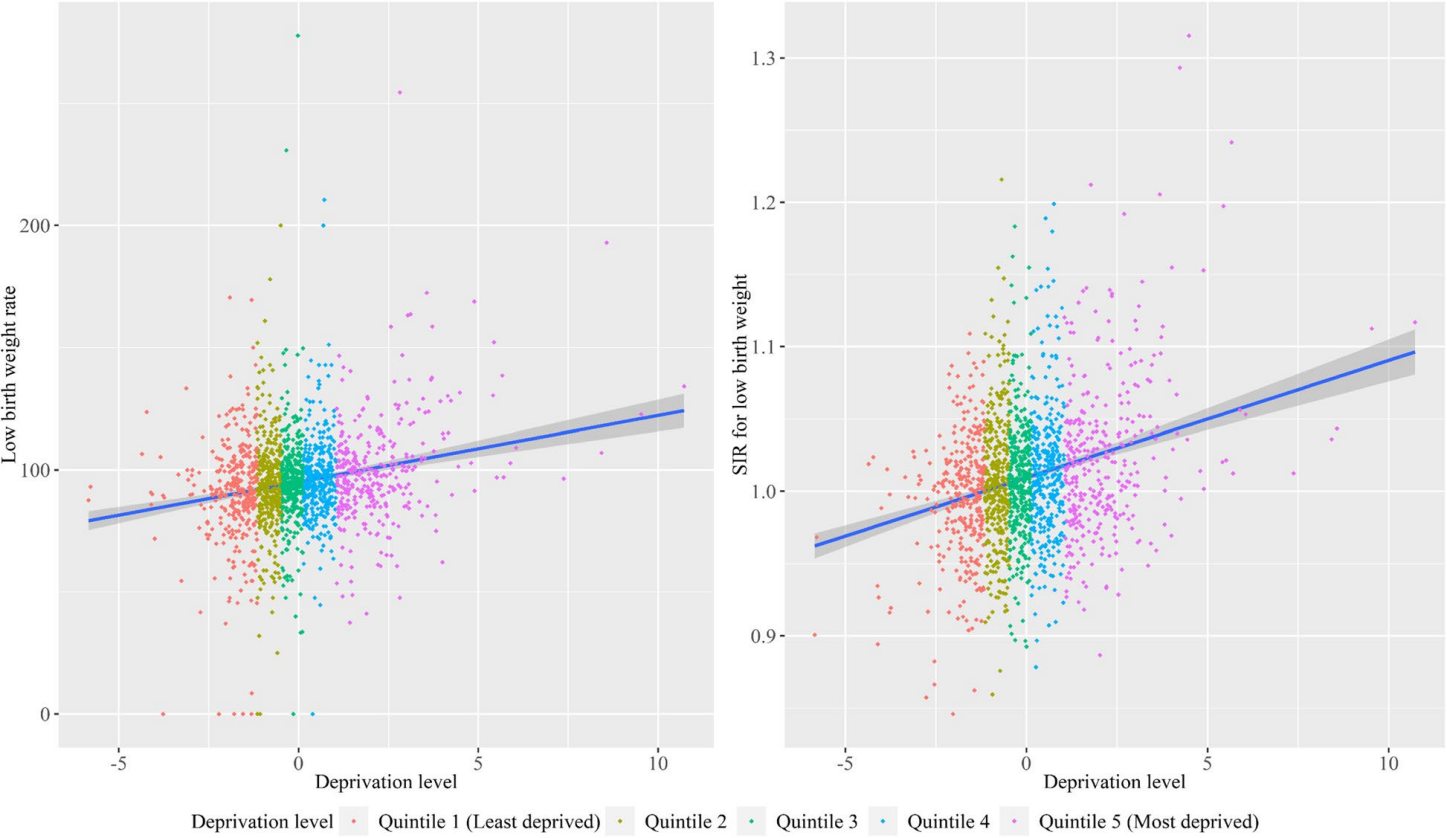
Scatterplots between the socioeconomic deprivation level and low birth weight (low birth weight rate and the SIR for low birth weight). SIR, standardized incidence ratio. Low birth weight rate indicates the number of low birth weight infants per 1,000 birth–years. The straight line in the figures indicates estimated values of the indicator by a linear regression model

Figure 2 depicts scatterplots between the socioeconomic deprivation level and low birth weight (low birth weight rate and the SIR for low birth weight). The correlation coefficient between the socioeconomic deprivation level and low birth weight rate was 0.196 (*p*-value < 0.001), and that between the socioeconomic deprivation level and the SIR for low birth weight was 0.260 (*p*-value < 0.001). Thus, there was a positive correlation.

Table 2 shows the low birth weight rate and other municipal characteristics depending on municipal socioeconomic deprivation levels. Low birth weight rates were observed to increase as the deprivation level increased. Additionally, the number of hospitals per capita tended to increase as the deprivation level increased. Investigations also revealed that the median number of medical clinics per capita in the most deprived areas was larger than in the least deprived areas.

**Table 2:** Low birth weight rate and other municipal characteristics depending on municipal socioeconomic deprivation levels

Characteristics	Quintile 1 (Least deprived, *n* = 338)	Quintile 2 (*n* = 337)	Quintile 3 (*n* = 337)	Quintile 4 (*n* = 337)	Quintile 5 (Most deprived, *n* = 338)
	Median (IQR)	Median (IQR)	Median (IQR)	Median (IQR)	Median (IQR)
Low birth weight rate^*^	91.1 (82.9–101.7)	93.1 (85.0 – 102.5)	94.2 (87.7 – 101.6)	95.7 (87.4 – 104.0)	97.7 (87.9 – 107.5)
Birth rate^†^	643.5 (493.7 – 815.8)	656.5 (521.7 – 781.5)	639.3 (525.7 – 755.4)	660.3 (537.4 – 775.7)	684.9 (563.2 – 820.9)
Proportion of women aged 15–49 years old (%)	17.9 (15.5 – 21.4)	18.4 (15.7 – 20.6)	17.8 (16.0 – 20.0)	18.3 (16.1 – 20.0)	17.9 (16.0 – 20.0)
Population density (population per hectare)	1.7 (0.4 – 14.6)	2.2 (0.4 – 8.2)	2.1 (0.7 – 6.6)	2.2 (0.8 – 7.0)	2.1 (0.8 – 8.0)
Number of physicians working in an obstetrics and gynecology department^†^	0.0 (0.0 – 6.4)	2.3 (0.0 – 7.1)	1.8 (0.0 – 7.3)	3.1 (0.0 – 8.2)	2.8 (0.0 – 8.6)
Number of medical clinics ^†^	65.0 (53.5 – 86.5)	68.6 (56.5 – 83.5)	67.8 (55.8 – 81.2)	68.7 (54.0 – 86.5)	70.6 (53.3 – 87.4)
Number of hospitals^†^	3.3 (0.0 – 6.7)	4.4 (0.0 – 8.1)	6.4 (3.4 – 9.7)	7.1 (3.8 – 10.6)	9.5 (5.2 – 13.6)

^*^Number of low birth weight infants per 1,000 birth-years

^†^Number per 100,000 persons

Table 3 shows results of the non-spatial Poisson regression and Bayesian spatial regression models. The relative risk increased as the deprivation level increased in the non-spatial regression model. However, the Moran’s I value for residuals of the regression model was 0.195 (*p* < 0.001), and it was suggested that it is better to use a spatial model in the analysis. The Bayesian spatial regression model showed a similar association, and a significant association was observed between the deprivation level and low birth weight.

**Table 3:** Results of non-spatial poisson regression and Bayesian spatial regression model

Variables	Non-spatial Poisson regression model		Bayesian spatial regression model
	Relative risk (95% confidence interval)	*p*-value	Relative risk (95% credible interval)
Deprivation level	1.028 (1.024–1.032)	< 0.001	1.030 (1.022–1.039)
Birth rate^*^	1.024 (1.018–1.031)	< 0.001	1.003 (0.992–1.015)
Proportion of women aged 15–49 years old	0.975 (0.967–0.983)	< 0.001	0.987 (0.972–1.002)
Population density	0.999 (0.997–1.001)	0.31	1.000 (0.992–1.008)
Number of physicians working in an obstetrics and gynecology department^*^	1.002 (0.998–1.006)	0.419	0.999 (0.994–1.005)
Number of medical clinics^*^	0.998 (0.993–1.002)	0.295	1.001 (0.994–1.009)
Number of hospitals^*^	0.989 (0.982–0.996)	0.002	1.001 (0.991–1.012)

^*^Number per 100,000 persons

## Discussion

An association between municipal socioeconomic deprivation level and the low birth weight in Japan was investigated, and a positive correlation was observed between them. In addition, a significant and positive association was identified for the association in the regression analysis. Here possible reasons for this phenomenon are discussed.

A possible reason for the association is that women with lower socioeconomic status tend to have risk factors associated with lower birth weight of their infants. Maternal smoking is a major risk factor associated with low birth weight in infants [36, 37]. In Japan, smoking prevalence tend to be high among persons with lower educational levels or lower household incomes [38, 39]. Psychosocial distress is also known to affect the risk of low birth weight in other countries [40, 41], and persons with lower socioeconomic status tend to be psychologically distressed in Japan [42]. Being overweight is another risk factor for low birth weight, and it increases the risk of pregnancy complications, such as gestational diabetes and preeclampsia [43]. In Japan, low household economic status was associated with being overweight among adolescents [44], and lower socioeconomic status was positively associated with higher body mass index [45]. Therefore, it is likely that regional differences in the proportion of women in lower socioeconomic status levels leads to regional difference in the rate of low birth weight.

Inadequate use of prenatal care was another possible reason for the association. Although the number of hospitals and medical clinics per capita was indeed large in the deprived areas, those medical cases might have been inadequately utilized in those deprived areas. A previous study suggested that prenatal care use effectively lowered the prevalence of low birth weight in a prefecture in Japan [46]. Other studies have indicated that lower socioeconomic status tends to be associated with less use of available prenatal care in other countries [47, 48]. Additionally, a higher rate of unplanned pregnancies among women with lower socioeconomic status has been suggested as a cause of lower prenatal care participation rates in France [47]. In Japan, lower high school enrollment percentages were associated with inadequate use of prenatal care across prefectures [49], and it is possible that the usage rate of prenatal care resources varied depending on the deprivation level. Furthermore, financial aids, which pregnant women can receive for prenatal care, may vary depending on the deprivation level because municipal tax revenues affect general finances in municipalities. General finances per capita are positively associated with public expenditures for prenatal care per pregnant woman in Japan [50]. Therefore, there may be some differences in the prenatal care that pregnant women can receive depending on the deprivation level.

An implication of this study is that differences in low birth weight rates existed which were dependent on municipalities and municipal socioeconomic deprivation level. It is known that inter-prefecture disparities in under-5 year old mortality widened over the decades in Japan, and increasing socioeconomic gradients are suggested as a possible reason [51]. There is a possibility that this regional disparity in low birth weight also affects regional differences in infant mortality or under-5 mortality. Additionally, it will be meaningful to investigate an association between participation rates in prenatal care or characteristics of perinatal medicine and deprivation level. If participation rates in prenatal care are low in deprived area, financial aid for healthcare costs or efforts aimed at increasing awareness of care options may be beneficial in those areas. Moreover, it is noteworthy that in Japan there are regional disparities in the number of physicians, and in the distribution of departments of obstetrics and gynecology [52]. If resources for antenatal care are insufficient in deprived areas, increasing the number of physicians or medical facilities engaging in antenatal care might be important. As the result of this study shows, the number of physicians working in an obstetrics and gynecology department per population is not small in deprived areas, but it is considered that an allocation of physicians to areas where low birth weight rates are particularly high is important in deprived areas. Community engagement activities to enhance information spread about prenatal care for pregnant women or support for pregnant women might also be effective [53].

A strength of this study is that Vital Statistics in all of Japan were used for the analysis. Therefore, the results indicated trends in all of Japan. Furthermore, although a previous epidemiological study showed a positive association between income and low birth weight in Japan [10], this study using nationwide data indicated that a lower socioeconomic status was positively associated with higher rates of low birth weight in Japan. Another strength is that we revealed geographical differences in the SIR for low birth weight in Japan using municipal-specific data. Hence, a more precise analysis could be conducted in this study compared with an analysis just using prefecture-level data.

There are some limitations. First, this was an ecological study, and an ecological fallacy might have existed in the presumed association. Second, it is not certain from this study whether the individual’s socioeconomic status, or the neighborhood’s deprivation was associated more with regional differences seen in low birth weight rates. Third, data on major factors associated with low birth weight, such as the gestational age, parity, fetal number, number of hospitals with departments of obstetrics and gynecology, could not be obtained for each municipality. Studies taking into account of preterm or term by gestational ages among low birth weight infants will be important in the future. Fourth, the mechanisms causing or influencing the association between deprivation and low birth weight in infants are not yet known. For example, it is not known whether the deprivation level increases preterm birth and/or intrauterine growth restriction. A study using individual data and examining various types of adverse birth outcomes will be important to resolve these limitations in the future.   

## Conclusions

We investigated an association between municipal socioeconomic deprivation level and low birth weight rate using aggregate national vital statistics data for all of Japan. As a result, there was a positive correlation between the deprivation level and SIR for low birth weight. In addition, a spatial regression analysis showed that there was a significant and positive association between the deprivation level and low birth weight.

## Data Availability

All the data used in this study are publicly available. Data sources are written in the Methods and References.

## Abbreviations

SIR: standardized incidence ratio
